# PKCδ as a Regulator for TGFβ1-Induced α-SMA Production in a Murine Nonalcoholic Steatohepatitis Model

**DOI:** 10.1371/journal.pone.0055979

**Published:** 2013-02-18

**Authors:** Su Jin Lee, Jeong Han Kang, Soo Young Choi, Ki Tae Suk, Dong Joon Kim, Oh-Shin Kwon

**Affiliations:** 1 School of Life Sciences, College of Natural Sciences, Kyungpook National University, Daegu, Korea; 2 Laboratory of Cell Biology, NCI, National Institutes of Health, Bethesda, Maryland, United States of America; 3 Department of biomedical Science and Research Institute for Bioscience and Biotechnology, Hallym University, Chunchon, Korea; 4 Department of Internal Medicine Hallym University College of Medicine, Chunchon, Korea; Rush University Medical Center, United States of America

## Abstract

The precise mechanism of TGFβ1 signaling in the progression of non-alcoholic steatohepatitis (NASH) has remained unclear. In particular, a potential regulatory mechanism by which PKCδ affects profibrogenic gene expression had never been explored. In this study, therefore, the role of PKCδ in TGFβ1 mediated α-SMA expression was investigated using NASH model mice. In preparation of the NASH model, male C57BL6/J mice were fed a methionine-choline-deficient (MCD) diet for 3 weeks, after which time they were intraperitoneally injected with lipopolysaccharide (LPS). In addition, Tlr4^Lps-d^ (CH3/HeJ) mice were used to demonstrate the TGFβ1 signaling’s dependency on TLR4 induction. Liver histology and hepatic hepatitis markers were investigated, and hepatic gene expression levels were determined by real-time PCR. Acute liver injury by LPS injection specifically elevated not only α-SMA expression but also phospho-PKCδ in this model. In contrast, Tlr4^Lps-d^ (CH3/HeJ) and blockade of TGFβ1 receptor by SB431542 resulted in a significant reduction of PKCδ activation and α-SMA expression. Moreover, the TGFβ1-induced α-SMA production was significantly reduced by a specific PKCδ inhibitor. These findings suggested that PKCδ plays a critical role in TGFβ1-induced α-SMA production in a NASH model. Thus, this was the first demonstration of the involvement of PKCδ in the regulation of α-SMA expression in NASH liver tissues, and the impaired induction of PKCδ phosphorylation by LPS in a steatohepatitis condition. Interestingly, treatment by PKCδ inhibitor caused dramatic reduction of myofibroblast activation, indicating that PKCδ represents a promising target for treating NASH.

## Introduction

Non-alcoholic fatty liver disease (NAFLD) is believed to be initiated by the accumulation of lipids in the liver (hepatosteatosis) [1], which may then progress to a clinical condition known as non-alcoholic steatohepatitis (NASH). NASH is characterized by inflammation, apoptosis of liver cells, and may [2], [3], [4] precise mechanism by which steatosis progresses to NASH is unknown, a “two-hit hypothesis” of the pathogenesis has been proposed to explain the progression of steatosis to NASH [5]. According to this hypothesis, steatosis represents the “first hit” or the first step, and various follow up “second hits” may in turn lead to the hepatocyte injury, inflammation, fibrosis and cellular damage characteristic of NASH [6]. The presumed factors initiating second hits include adipocytokines, pro-inflammatory cytokines (*e.g.* TNF α), oxidative stress and subsequent lipid peroxidation. In addition, it should be noted that exposure to endotoxins or lipopolysaccharides (LPS) can also act as a second hit resulting in progressive liver injury.

The fatty liver is highly sensitive to LPS, and Toll-like receptor 4 (TLR4) serves as a specific receptor for LPS [7]. TLR4 impacts the regulation of innate immune responses by provoking inflammatory responses to exposure by LPS. A recent work has implicated TLR4, as expressed on hepatic stellate cells (HSCs), as a key driver of liver fibrosis, owing to the stimulatory effect of TLR4 activation on the transforming growth factor β1 (TGFβ1) pathway [8]. Activated myofibroblasts play a critical role in the wound-healing process and the progression of NASH. Myofibroblasts can be derived from hepatocytes, cholangiocytes, portal fibroblasts and fibrocytes as well as activated HSCs [9]. Expression of α-SMA, the actin isoform characteristic of vascular smooth muscle cells, is a marker of myofibroblast differentiation. Myofibroblasts secrete TGFβ1 in an autocrine fashion, which stimulates the myofibroblast as part of the processes of wound healing and tissue fibrosis. TGFβ1 is a key fibrogenic cytokine and disruption of TGFβ1 homeostasis is linked to enhanced fibrogenesis [10].

Protein kinase C (PKC) is a family of serine/threonine protein kinases that play a central role in various cellular activities such as the control of growth, differentiation, and apoptosis. Depending upon their co-factor requirements, PKC members are classified into conventional (α, β and γ), novel (δ, θ and ε) and atypical subfamilies. It has been suggested that novel PKC isoforms play a role in the development of fat-induced pathological conditions. Interestingly, novel PKC isoform activation mediated by free fatty acids appeared to be related to an increase in diacylglycerol [11]. In addition, the PKC family members are considered key signaling mediators in the process of inflammation. In particular, novel PKC isoenzymes may be associated with tissue injury and various inflammatory responses. Indeed, the role of PKCδ in inflammation and immunity has been recently confirmed using PKCδ–deficient mice [12]. These findings gave rise to hypothesis that PKCδ activation is involved in the progression of NASH. However, a causative link between the activation of PKCδ and the pathology of NASH remains to be elucidated.

Therefore, the major objective of the present work was to determine the role of PKCδ in the development of NASH. The involvement of the PKCδ signaling pathway in the regulation of α-SMA expression was explored in mice fed a methionine-choline-deficient (MCD) diet and injected with LPS, with an emphasis on assessment of the TGFβ1 signaling pathway. MCD dietary rodent model is a widely used nutritional model of NASH with histological features that most closely resemble those seen in humans. The aim of this study was to determine the role of PKCδ in TGFβ1 signaling pathway and thus enhances α-SMA production. Our findings suggested that utilizing PKCδ is a promising strategy for the treatment of NASH.

## Materials and Methods

### Animal Models and Experimental Protocol

Male C57BL/6 and Tlr4^Lps-d^ (CH3/HeJ) mice of 8 weeks of age were purchased from Orient Bio, Inc. (Seoul, Korea). The animals were housed in a temperature- and humidity-controlled room and subjected to a daily cycle of 12 h of light and 12 h of darkness. Initially, all mice were acclimated to the vivarium for at least one week prior to use in experiments. We prepared an MCD diet which contained corn oil and sucrose [40% (w/w) fat and 40% (w/w) carbohydrate]. When the mice reached 9 weeks of age they were fed either the MCD or control diet for 3 weeks. Then, the mice were injected intraperitoneally (i.p.) with either a single dose of 2.5 mg/kg LPS (Sigma Chemical, St. Louis, MO) or normal saline. The δV1-1 (PKCδ inhibitor, [C-SFNSYELGSL]) was synthesized and conjugated to TAT-carrier peptide (amino acids 47–57) via a disulfide conjugation through cysteines at the N-terminus. SB431542, Rottlerin, TAT or δV1-1 was injected intraperitoneally 1 h, respectively, prior to LPS treatment. All animals were sacrificed after 6 h in order to obtain serum and liver tissue samples for analysis. The mice were fed a ND group (n = 8), MCD group (n = 8) or LPS–injected MCD group (n = 9).

### Immunohistochemistry

Formalin-fixed and paraffin-embedded sections were used in the immunohistochemistry analysis in this study. The primary antibodies applied to the sections were rat antibody against F4/80 (dilution 1∶500; Serotec, Oxford, UK), a rabbit antibody against α**-**SMA (dilution 1∶400, Epitomics, Burlingame, CA), a rabbit antibody against mouse phospho-PKCδ (Thr505) (dilution 1∶200, Abcam, Cambridge, MA), or a rabbit antibody against E-cadherin (dilution 1∶200, Cell Signaling Technology Inc, Beverly, MA). The sections were incubated with the appropriate secondary antibodies, and the immunoreactive products were visualized using a DAB color reagent and hematoxylin counterstain.

### Real-time Quantitative Reverse Transcription Polymerase Chain Reaction

Real-time (RT) PCR analysis was performed using the primers shown in Table S1. The total RNA was isolated from the mice livers using TRIzol (Invitrogen) according to the manufacturer’s directions. Quantitative RT-PCR was performed using SuperScript III RNase H^−^ (Invitrogen). PCR amplification was performed on a LightCycler (Roche Diagnostics, Castle Hill, Australia) using SYBR Green I as a double-stranded DNA-specific binding dye and continuous fluorescence monitoring. Relative quantification of the target mRNA expression was calculated and normalized to the expression of 18S. The results, based on the ratio of target mRNA to 18S amplification, were presented as the fold induction in mRNA expression relative to the amount observed in the control samples.

### Western Blot Analysis

Proteins from lysated liver tissue were analyzed by Western blotting. The membrane was exposed to specific polyclonal antibodies against α**-**SMA(1∶1000, Epitomics), vimentin (1∶1000, Abcam), PKCδ (1∶1000, Cell signaling), phospho-PKCδ (Thr-505) (1∶1000, Abcam), phospho -IκBα (Ser-32/36) (1∶1000, Cell signaling) and E-cadherin antibody (1∶1000, Cell signaling). Nonspecific antibody binding was blocked using 5% bovine serum albumin (BSA) in TBS-T. The blots were incubated with primary antibody in block solution for 2 h at room temperature and subsequently washed 3 times in TBS-T. The appropriate secondary antibody was added and then incubated for 1 h at room temperature. After washing 3 times with TBS-T an enhanced chemiluminescence (ECL) detection system was applied for band visualization. The densities of the bands were quantified using the Quantity One 1D image analysis software program (Bio-Rad). The anti-GAPDH antibody (1∶1000, Santa Cruz) was used as a loading control.

### Statistical Analysis

Analysis was performed using standard statistical software (SPSS for Windows, version 17.0). The data are presented as mean ± SEM and the comparison between groups was made using one-way ANOVA followed by the Student-Newman-Keuls test. *P<0.05 was considered statistically significant. Data were presented as bar graphs including the mean ± standard deviations (SD) of at least three independent experiments.

## Results

### Hepatic Steatosis in Mice fed MCD Diet

The MCD diet mice resulted in subsequent hepatic histology observations similar to that observed in human cases of NASH [13]. Feeding the mice an MCD diet for 3 weeks induced progressive weight loss, significant increase in lipid accumulation and infiltration of inflammatory cells in the liver (Fig. S1A). Hepatic TG concentration dramatically increased in the MCD fed mice and their transaminase levels were markedly elevated compared to the normal diet (ND) mice. Administration of LPS to the MCD mice induced a more significant increase in serum ALT and AST levels.

**Figure 1:**
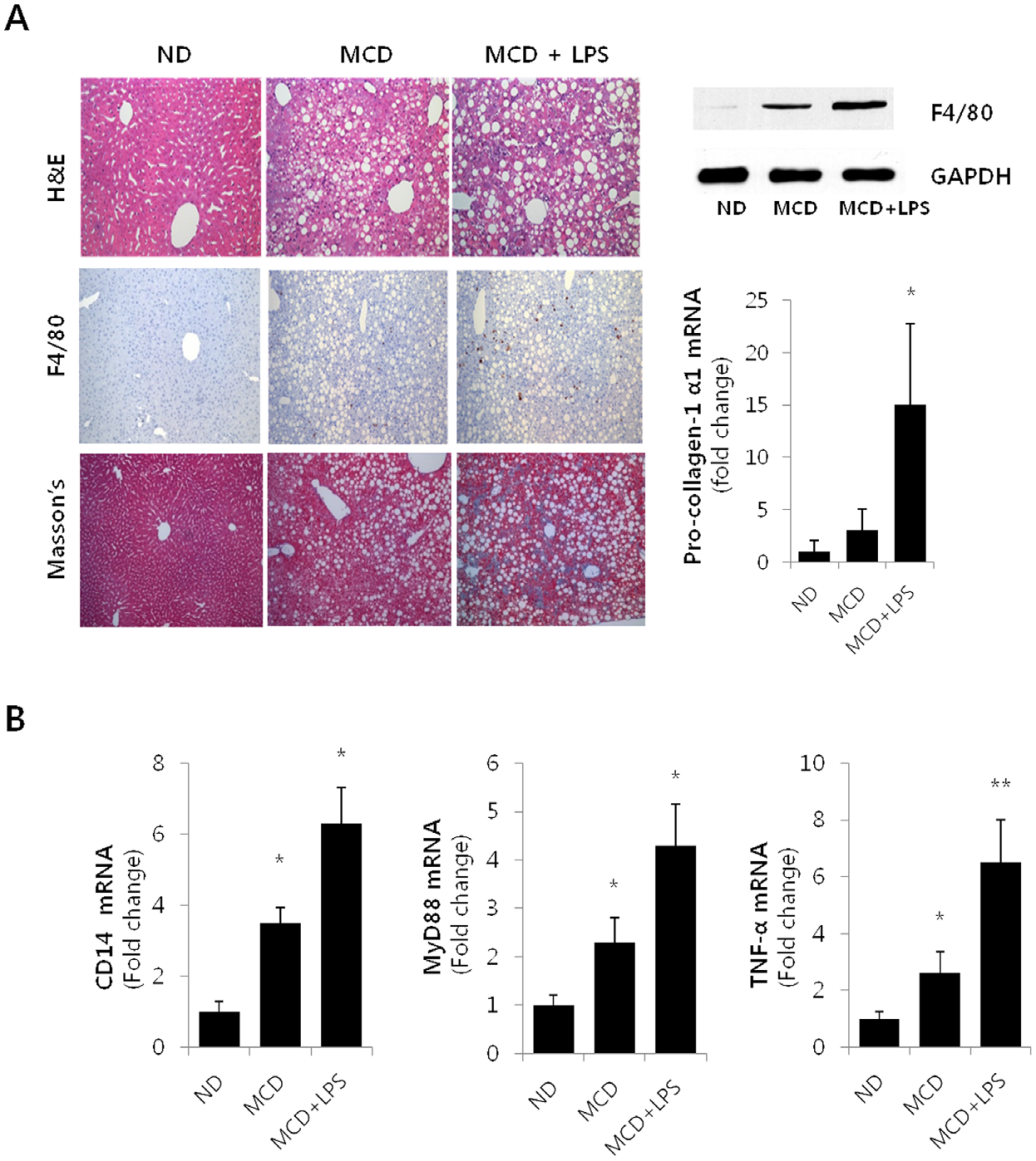
Hepatic Steatosis in LPS-injected MCD diet mice. (A) Top panels on the left show histology of liver sections from a ND, MCD and LPS-injected MCD mice by Hematoxylin-eosin (H&E) staining (original magnification 200×). Macrophage infiltration was determined by immunohistochemistry analysis of F4/80 (medium panel), and progressive fibrosis was detected by Masson’s trichrome (Masson’s) stain (bottom panel). The right panels, Western blot (top) and qRT-PCR (bottom) analyses were used to quantitate the expression of hepatic F4/80 and hepatic mRNA levels for *col1α1*, respectively. For the densitometric analysis, *n* = 3; ^*^ *P*<0.05. (B) Hepatic levels of *CD14, Myd88 and TNF-α* mRNA were measured by qRT-PCR in ND, MCD and LPS-injected MCD mice. Genes were normalized to18S rRNA as an internal standard and data are shown as fold increase. *P<0.05, **P<0.01.

Liver histology of the MCD animals revealed moderate, nonzonal macrovesicular lipid accumulation and moderate lobular necroinflammation (Fig. 1A, top/right panel). LPS-injected MCD diet mice were observed to have higher inflammatory activity as compared to the saline-injected MCD diet mice. In order to characterize the phenotype of the macrophages that accumulated in the liver during the LPS induced hepatotoxicity, an immunohistochemical study was performed using antibodies against F4/80, a marker of mature macrophages (medium panel). Hepatic macrophage accumulation increased in mice fed the MCD diet as compared to the control diet mice. Indeed, LPS treatment (2.5 mg/kg intraperitoneally) led to a marked elevation in the number of F4/80-positive cells. As shown in the bottom panel, the Masson’s stained liver histology section showed that progressive fibrosis occurred in the steatohepatitis model. LPS-injected MCD diet mice showed an increase in central/pericentral vein fibrosis and portal tract fibrosis when compared to the ND and MCD diet mice. Changes in F4/80 protein content were also quantified by Western blot using total liver protein (Fig. 1A. upper/right panel), and collagen α1(I) mRNA levels, as determined by RT-PCR analysis, were observed to significantly increase in the LPS-injected MCD mice as compared to the ND mice (bottom/right panel).

Toll-like receptors (TLRs) may be the key receptors controlling inflammation, myofibroblast accumulation and fibrosis in the liver. LPS-mediated TLR4 activation also plays a role in fibrogenic signal pathways and HSC activation. In order to explore TLR4-dependent profibrogenic effects, RT-PCR analysis of liver homogenates was performed (Fig. 1B). The co-receptor, CD14, and the adapter MyD88 in the TLR4 receptor complex significantly increased as determined by mRNA levels in the LPS-injected MCD diet mice [14]. Similarly, the levels of the fibrogenesis mediators TNFα showed significant increase in the LPS-injected MCD mice.

### LPS Stimulated PKCδ Activation in MCD Diet Mice

**Figure 2:**
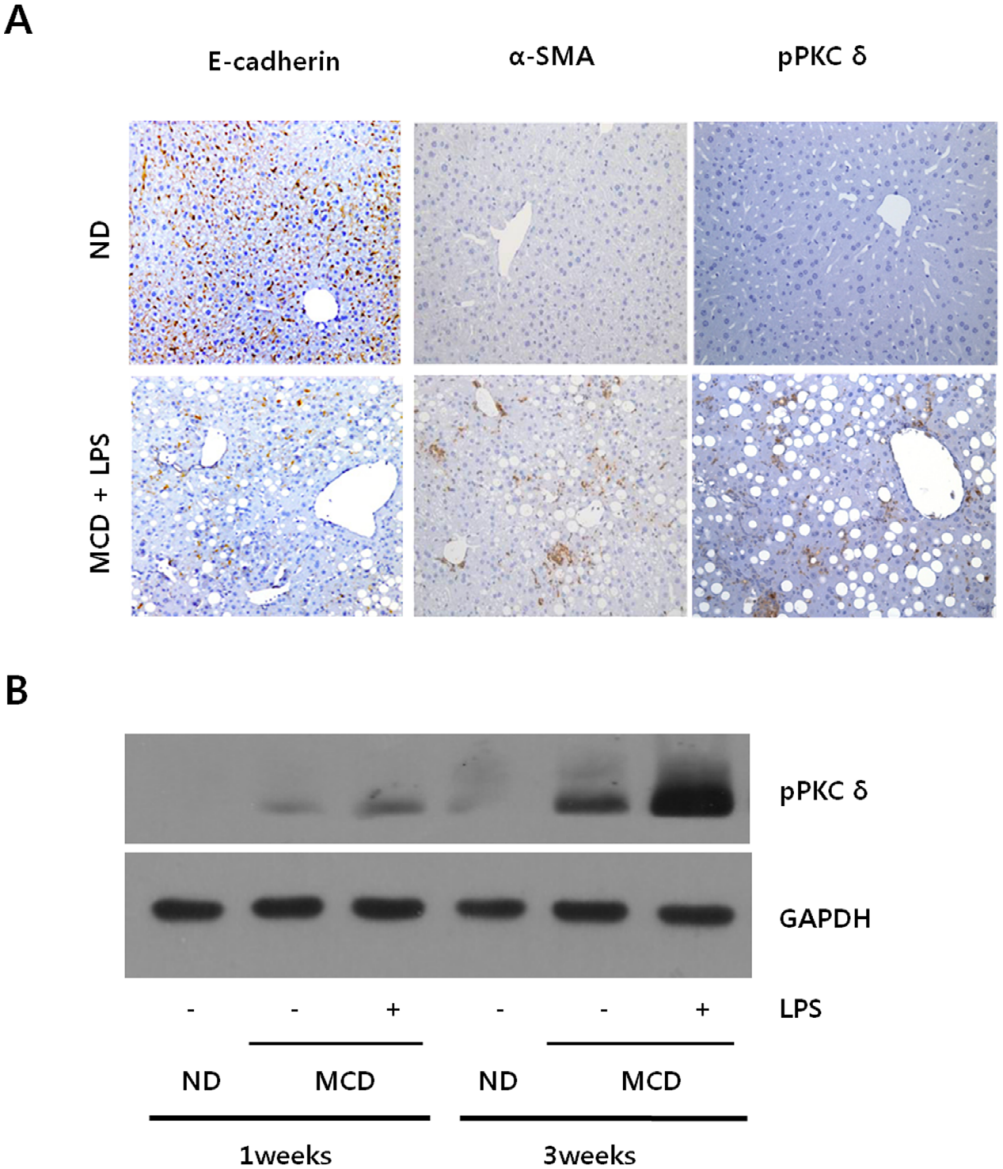
LPS-stimulated PKCδ activation in MCD diet mice. (A) Liver sections from ND mice and LPS-injected MCD mice were analyzed for E-cadherin, α-SMA and phospho-PKCδ expression using immunohistochemistry staining (original magnification 200×). (B) Liver tissue extracts were prepared from mice fed MCD diet for 1 weeks or 3 weeks and subsequently injected with 2.5 mg/kg LPS. Liver phospho-PKCδ protein contents were quantified by Western blot using an equal amount of total liver proteins. Expression levels were normalized relative to GAPDH. Liver extracts from a pool of three mice in each group were analyzed by Western blot using specific antibodies.

We then investigated the PKCδ mechanisms by which LPS induced liver injury in MCD fed mice. LPS-injected MCD mice showed increases in α-SMA, a marker for activated stellate cells and myofibroblasts. As expected, the immunohistochemistry analyses of the liver tissues showed a reciprocal correlation between ECAD expression and α-SMA (Fig. 2A). Interestingly, hepatic phospho-PKCδ also dramatically increased in the LPS-injected MCD diet mice as compared to the MCD diet mice injected with saline. It must be noted that the expression pattern of phospho-PKCδ was very similar to that observed in α-SMA. These findings resulted in the hypothesis that PKCδ activation correlates with α-SMA production.

These the immunohistochemistry results were confirmed by Western blot. As shown in Fig. 2B, the temporal variation of phospho-PKCδ level was examined. The differences in the phospho-PKCδ levels became noticeable after 1 week of the diet and became more significant after 3 weeks. However, practically no obvious activation was detectable in ND mice for the specified times. Moreover, LPS-injected MCD diet mice were observed to have about two-fold higher phospho-PKCδ level as compared to the MCD diet mice injected with saline.

### Correlation between PKCδ Activation and TGFβ1-induced α-SMA Expression

**Figure 3:**
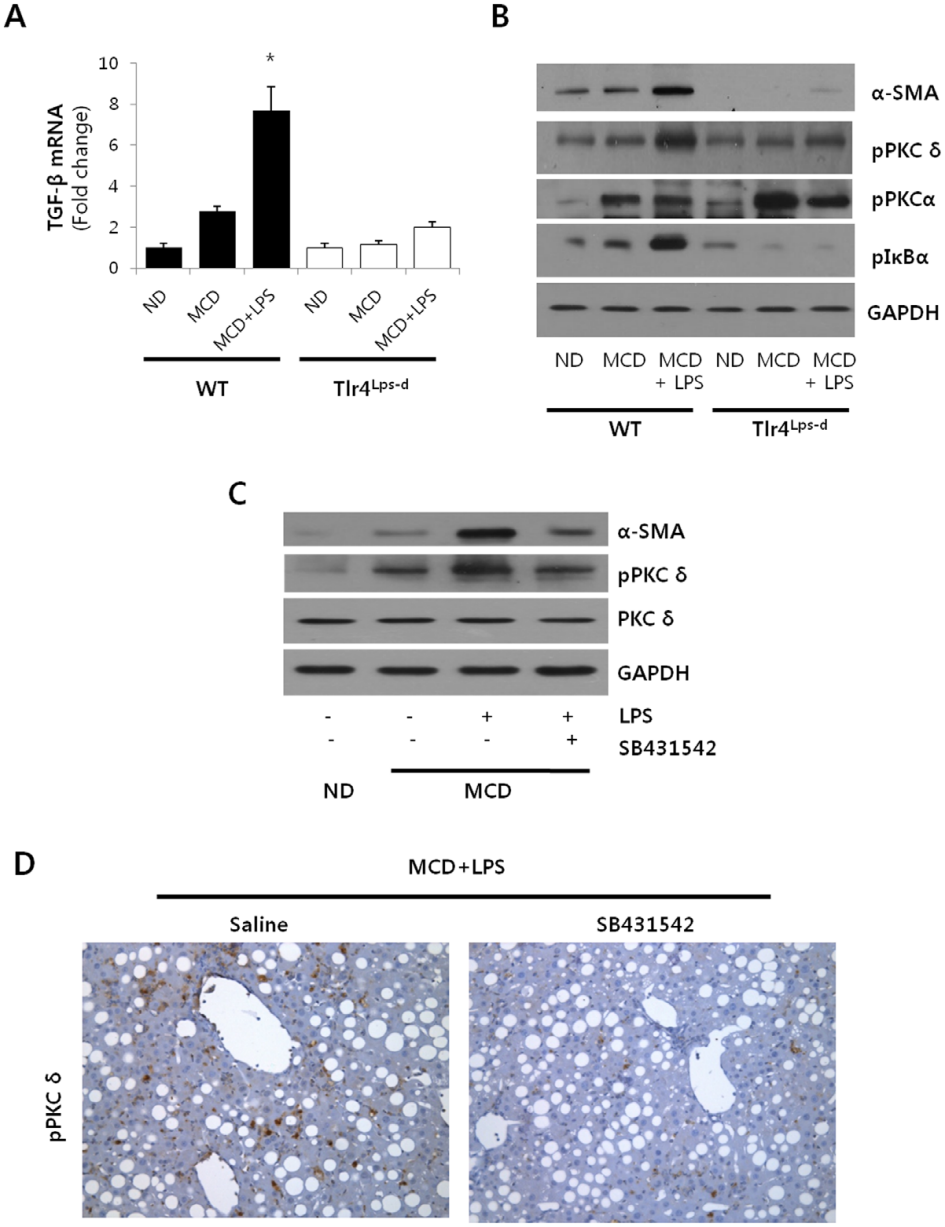
Involvement of PKCδ in TGFβ1-induced α-SMA expression. (A) Hepatic levels of *TGF β1* mRNA from ND, MCD and LPS-injected MCD mice in WT or Tlr4^Lps-d^ (CH3/HeJ) were measured by qRT-PCR. Genes were normalized to18S rRNA as an internal standard. *P<0.05 versus the ND group. (B) Mice were injected with 2.5 mg/kg LPS for 6 hours in Tlr4^Lps-d^ (CH3/HeJ) and WT. Liver α-SMA, phospho-PKCδ, phospho-PKCα and phospho-IκBα protein contents were quantified by Western blot. Expression levels were normalized relative to GAPDH. Liver extracts from a pool of three mice in each group were analyzed by Western blot using specific antibodies. (C) Liver tissue extracts were prepared from mice fed MCD diet for 3 weeks and subsequently injected with 2.5 mg/kg LPS and TGFβ1 inhibitor (SB431542). Liver α-SMA, phospho-PKCδ and PKCδ protein contents were quantified by Western blot using an equal amount of total liver proteins. Liver extracts from a pool of three mice in each group were used. (D) Representative liver tissue sections from LPS-treated MCD mice injected with either saline or SB431542 (10 mg/kg) were stained with phospho-PKCδ antibody (original magnification 200×).

To understand the role of endogenous TLR4 in mediating the LPS-induced PKCδ activation in NASH, we examined the TGF β1, α-SMA expression and PKCδ activation in response to LPS from mice that carry a spontaneous mutation in TLR4 (Tlr4^Lps-d^) [15]. As shown in Fig. 3A, TGFβ1 was significantly induced in the LPS-injected MCD diet mice, where the Tlr4^Lps-d^ (CH3/HeJ) mice resulted in a significant decrease in TGF β1 expression as determined by mRNA levels. To verify the activation state of hepatic membrane PKCδ, we determined phosphorylated PKCδ at Thr^505^, which is required for full activation. Similarly, the association of PKCα activation and membrane translocation was further evidenced by measuring phosphorylation of PKCα. We also observed a significant up-regulation of PKCδ activation in liver isolated from WT mice after LPS injection but not in those from Tlr4^Lps-d^ (CH3/HeJ) mice, suggesting that TLR4-dependent up-regulation of PKCδ is an integral part of the HSC activation process *in vivo* (Fig. 3B). However, Tlr4^Lps-d^ (CH3/HeJ) mice failed to block the phospho-PKCα expression. These results indicated that PKCδ could be involved specifically in TGFβ-induced α-SMA expression. Similarly, LPS cause to the enhancement of phospho-IκBα and α-SMA expression in WT but not Tlr4^Lps-d^ (CH3/HeJ) mice.

LPS regulated TGFβ1-induced signals from HSC in TLR4/MyD88 in a dependent manner, thus modulating liver fibrosis in NASH. Moreover, the MCD-induced TGFβ1 may contribute to HSC activation, and α-SMA production, a hypothesis which was examined by immunoblot analysis (Fig. 3C). As expected, α-SMA and phospho-PKCδ dramatically increased in the LPS-injected MCD diet mice as compared to the MCD diet mice injected with saline. Moreover, PKCδ activation might occur in a TGFβ1 dependent manner. Pretreatment with SB431542, a TGFβ1 receptor inhibitor, was observed to down-regulate α-SMA expression and attenuate the phospho-PKCδ production. In order to confirm if TGFβ1 inhibition was accompanied by changes in PKCδ activity, liver sections were stained with phospho-PKCδ antibody (Fig. 3D). Pretreatment with SB431542 significantly reduced phospho-PKCδ levels in LPS-injected MCD diet mice as compared to the LPS-injected MCD diet mice treated with saline.

### Reduction of Hepatic Fibrosis by the Regulation of PKCδ

**Figure 4:**
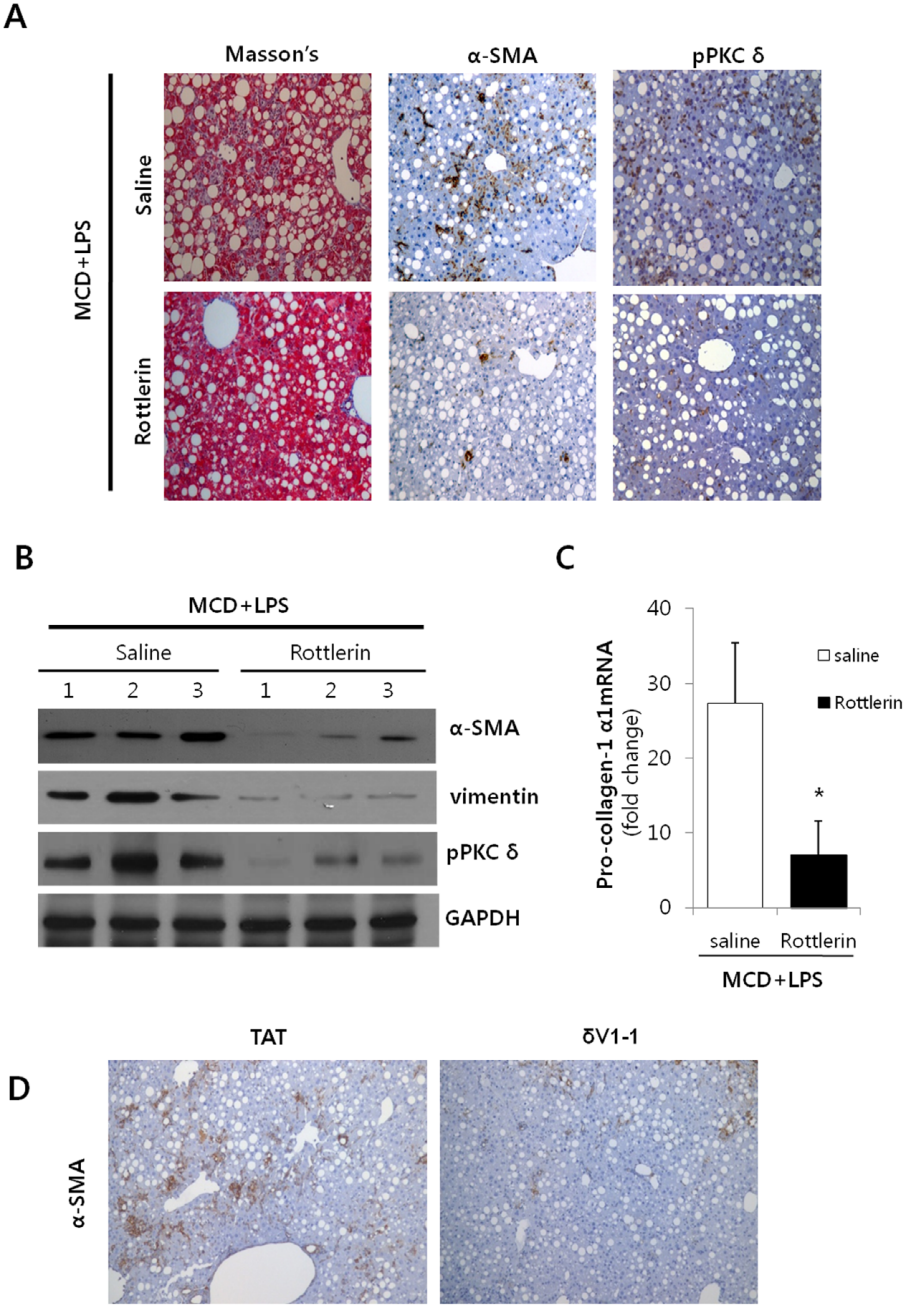
Effects of Rottlerin on LPS-injected MCD mice. (A) Representative liver tissue sections from LPS-treated MCD mice injected with either saline or Rottlerin (10 mg/kg) were stained with Masson’s trichrome (Masson’s) and α-SMA and phospho-PKCδ antibody (original magnification 200×). (B) Liver tissue extracts were prepared from mice fed the MCD diet for 3 weeks and subsequently injected with 2.5 mg/kg LPS and 10 mg/kg Rottlerin. α-SMA, vimentin and phospho-PKCδ protein content were quantified by Western blot using equal amounts of total liver proteins. The levels of α-SMA and vimentin were observed to significantly decrease in the Rottlerin treatment group. For statistical significance, three liver extracts from each individual were used for each group. Expression levels were normalized relative to GAPDH. (C) mRNA levels of hepatic collagen were determined by qRT-PCR analysis. Data plots represent the mean ± SD of three independent experiments. *P<0.05 versus saline group. (D) Indicated LPS-injected MCD diet mice were pretreated with TAT or δV1-1 peptide (0.2 mg/kg) for 1 hour. Representative liver tissue sections from LPS-treated MCD mice injected with either TAT or δV1-1 peptide were stained α-SMA with antibody (original magnification 200×).

Recent observations have linked the progression of steatohepatitis to the stimulation of the TGFβ1 dependent signaling pathway in hepatocytes upon injury [16], [17]. Thus, we hypothesized that the reduction of myofibroblasts and their associated proinflammatory cytokines could reduce the development of NASH, *in vivo*. In order to test whether or not PKCδ activation is implicated in hepatic fibrogenesis, Rottlerin was pretreated into LPS-injected MCD diet mice. Fibrosis was induced in the LPS-injected MCD diet mice, whereas the Rottlerin preinjected mice resulted in a significant decrease in fibrosis as demonstrated by trichrome staining (Fig. 4A). Simultaneously, α-SMA and phospho-PKCδ expression dramatically decreased in the MCD/Rottlerin mice as compared to the LPS-injected MCD diet mice. For the purpose of further demonstration, Western blot analyses were performed in order to compare the expression of α-SMA and vimentin (Fig. 4B). The levels of α-SMA and vimentin were observed to significantly decrease in the Rottlerin treatment group. Similarly, the α-SMA and vimentin levels were reduced by general PKC inhibitor, Gö6983 (Fig. S2A). The reduction of *pro-collagen-1α1* mRNA expression, an early marker of fibrogenesis, was demonstrated by RT-PCR (Fig. 4C). To validate the inhibition effect, the peptide δV1-1, PKCδ inhibitor, was pretreated LPS-injected MCD diet mice. As expected, the δV1-1 preinjected mice resulted in a significant decrease α-SMA expression in LPS-injected MCD diet mice as compared to the LPS-injected MCD diet mice treated with TAT. Again, this effect was confirmed using either SB431542 or Gö6983 (Fig. S2B). Conclusively, inhibiting hepatic PKCδ activity with Rottlerin may reduce NASH related fibrogenesis, despite exerting no net benefit on hepatocellular injury.

## Discussion

The pathogenesis of NASH, as caused by metabolic factors, is poorly understood at this time. However, many studies have pointed to the possibility that severe lipid accumulation is an essential precondition. Steatohepatitis caused by lipogenic MCD appears to trigger development of hepatic steatosis, ER stress, focal inflammation, hepatocyte necrosis and fibrosis [13], [17], [18], but simple hepatosteatosis may not trigger such events. The pathological changes associated with an MCD diet could be further amplified by the presence of additional signals such as LPS. Interestingly, LPS stimulation enhances the phosphorylation levels of PKC isoforms in murine macrophage RAW264.7 cells [19]. LPS stimulates TGFβ1 signaling from HSC through TLR4 [20], [21]. It is well known that TGFβ1 is a key profibrogenic cytokine. Increased cytokine distinguishes NASH from NAFLD, and this is thought to contribute to the increased risk for liver fibrosis in NASH. Thus, TGFβ1 plays roles as an activator and survival factor for HSCs, and subsequently regulates gene induction promoting ECM deposition. After injury, for instance, HSC trans-differentiates into proliferating myofibroblast-like cells that produce abundant levels of fibrillar collagen [22]. Myofibroblasts play a critical role in the wound healing process and pathological organ remodeling [23]. However, the relationship between the LPS-activated PKC signal pathway and myofibrosis in NASH remains unknown.

Numerous studies have implicated TGFβ1 and PKC as mediators of ECM accumulation [24], [25], [26]. In particular, the involvement of PKCδ in the TGFβ1 dependent regulation of ECM components has been proposed [27]. Recently, it has been shown that PKCδ is specifically activated by TGFβ1 in human mesangial cells and plays a role in collagen I accumulation via the Smad pathway [28]. Another study has demonstrated that TGFβ1 induced PKCδ activation regulates Smad3 activity resulting in transcriptional induction of *COL1A*2 and fibronectin [29]. Moreover, PKCδ has been shown to be essential in TGFβ1-induced fibronectin [29] and elastin synthesis in lung fibroblast [30]. These results indicated that TGFβ1 could stimulate ECM accumulation in several cell types by modulating PKCδ activity.

In this work, LPS was administered to MCD diet mice by intraperitoneal injection in order to evaluate the pathogenesis of NASH. Our working model assumed that the PKCδ signal pathway induced by TGFβ1 stimulation plays a critical role in α-SMA production in NASH. Thus, we demonstrated the involvement of PKCδ in TGFβ1-stimulated α-SMA expression using specific pharmacological inhibitors. Indeed, the inhibition of TGFβ1 receptor by SB431542 and Tlr4^Lps-d^ mice actually blocked PKCδ activation and α-SMA expression in NASH. Moreover, pre-injection with the novel-type PKCδ inhibitor, Rottlerin, dramatically reduced LPS-induced α-SMA and collagen levels. It should also be pointed out that PKCδ activation levels in tissues had never been previously explored (Fig. 2A). As such, our data clearly outlined, not only the predominant role that PKCδ plays in TGFβ1-mediated α-SMA expression in NASH, but also that the exogenous administration of PKCδ inhibitor sufficiently blocked the TGFβ1 pathway and the effects of fibrosis. Therefore, we have demonstrated that LPS stimulates PKCδ activation through TGFβ1 signaling, and that PKCδ subsequently further amplifies fibrosis in NASH. Although the PKCδ is shown to play a role in TGFβ1 induced fibrosis, other cellular mechanisms should also be considered. For example, PKCδ is also known to regulate production of nitric oxide, insulin signaling, as well as expression of inflammatory cytokines, all of which could indirectly influence α-SMA expression [31], [32], [33], [34]. Thus, further experiments are needed to differentiate these possibilities.

We mainly focused our effort on determining the role of the PKCδ activation in NASH. Our results pointed to several novel characteristics of PKCδ in a murine NASH model. First, we noted that the LPS-induced TGFβ1 signal pathway was mediated, at least in part, by PKCδ activation. In the TGFβ1 signaling pathway, a potential regulatory mechanism by which PKCδ affects profibrogenic gene expression, such as observed in α-SMA, had never been explored. Thus, our data demonstrated for the first time that PKCδ activation is involved in α-SMA expression in MCD diet mice, suggesting a novel role for PKCδ in stimulating myofibroblasts. Second, an exciting finding from the present study was that blockage of the PKCδ pathway caused significant regression of the impact of hepatic fibrosis. Our *in vivo* study showed that treatment using the PKCδ inhibitor may prevent myofibroblast activation. The discovery of this antagonistic role may lead to development of new therapeutic approaches for the treatment of fibrotic diseases.

Although it is known that myofibroblasts may play a central role in NASH, the molecular mechanisms involved in the progress remain unclear. In this study, we observed that LPS significantly increased hepatic TGFβ1 and phospho-PKCδ production in MCD diet mice resulting in the enhancement of α-SMA expression. Thus, to the best of our knowledge, we demonstrated for the first time that PKCδ activation is strongly stimulated by TGFβ1 in a NASH liver model and thus, activated PKCδ is involved in the regulation of α-SMA production. In addition, treatment with the PKCδ inhibitor prevented fibrogenesis with dramatic results in LPS-treated MCD diet mice. As far as we know, this was the first analysis in which PKCδ activation was shown to exhibit a positive correlation with the development of NASH. Therefore, although the detailed regulation mechanism needs to be further investigated, the antagonistic role of the PKCδ inhibitor may open new possibilities in controlling the pathological effects of TGFβ1 signaling in fibrotic diseases.

## Supporting Information

Figure S1 **Effect of MCD diet and LPS on weight loss and hepatocellular damage.** (A) Hepatic triglyceride, ratio of liver to body weight, ALT and AST values were analyzed in mice fed normal diet, MCD diet, or MCD diet and intraperitoneally injected with LPS (2.5 mg/kg). Mean SE of results from controls (n = 5) and each MCD diet-fed group (n = 8–9/group).
(TIF)

Figure S2 **Effect of TGFβ1** **inhibitor in LPS-injected MCD mice.** (A) Liver tissue extracts were prepared from LPS-treated MCD mice injected with buffered saline or Gö6983 (10 µM), and then the vimentin and α-SMA levels were evaluated by immunoblot. For evaluation of statistical significance, three liver extracts from each individual were used for each group**.** (B) mRNA levels of hepatic collagen was determined by qRT-PCR analysis. Data plots represent the mean ± SD of three independent experiments; *P<0.05.
(TIF)

Figure S3 **Colocalization of α-SMA and phospho-PKCδ in NASH.** The LPS-injected MCD diet mice express α-SMA (*red*) and phospho-PKCδ (*green*) were assessed for colocalization by confocal microscopy. Yellow fluorescence in the merged image presents co-localization of α-SMA and phospho-PKCδ (Original magnification, 400×).
(TIF)

Table S1 **Primers used for real time PCR.**
(DOCX)
