# Supplementary figures and images for: PKCδ as a Regulator for TGFβ1-Induced α-SMA Production in a Murine Nonalcoholic Steatohepatitis Model
Source: PLoS One. 2013 Feb 18;8(2):e55979. doi: 10.1371/journal.pone.0055979 (PMC3575342; doi:10.1371/journal.pone.0055979)

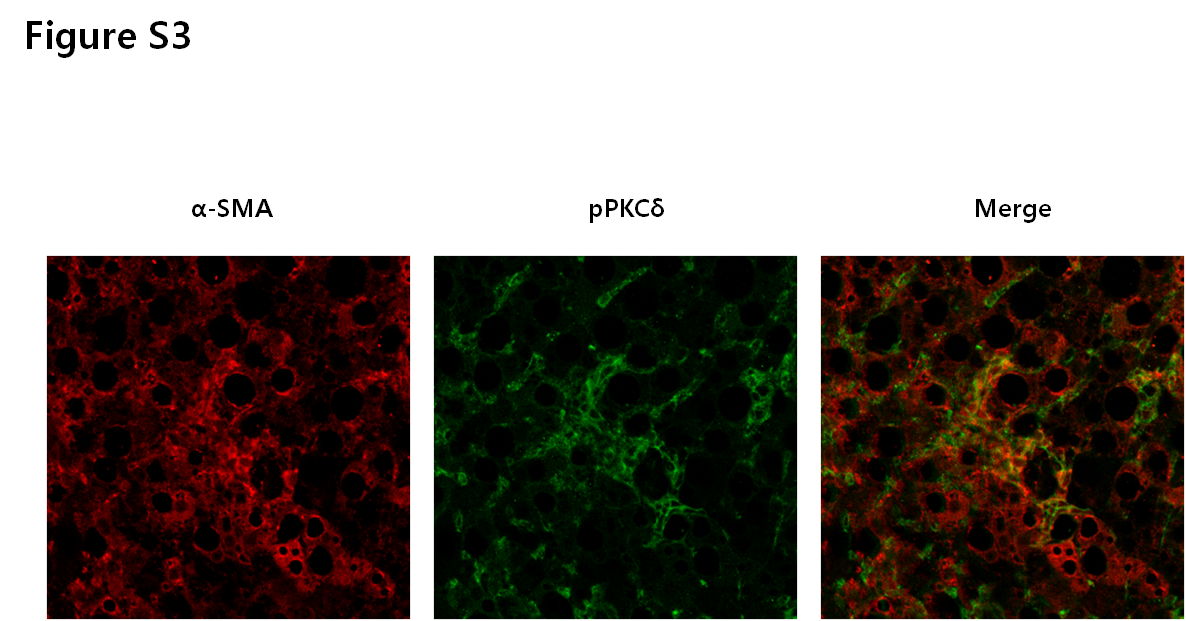

Supplement: Figure S3 — Colocalization of α-SMA and phospho-PKCδ in NASH. The LPS-injected MCD diet mice express α-SMA (red) and phospho-PKCδ (green) were assessed for colocalization by confocal microscopy. Yellow fluorescence in the merged image presents co-localization of α-SMA and phospho-PKCδ (Original magnification, 400×). (TIF) [file pone.0055979.s003.tif]
