# Supplementary material for: PKCδ as a Regulator for TGFβ1-Induced α-SMA Production in a Murine Nonalcoholic Steatohepatitis Model
Source: PLoS One. 2013 Feb 18;8(2):e55979. doi: 10.1371/journal.pone.0055979 (PMC3575342; doi:10.1371/journal.pone.0055979)
Supplement: Table S1 — Primers used for real time PCR. (DOCX) [file pone.0055979.s004.docx]

**Supplementary information**

**Table. S1 : primers used for real time PCR**

| **Primer name species** |  | **sequence** |
| --- | --- | --- |
| 18S Mouse |  | (F)5'-GTA ACC CGT TGA ACC CCA TT-3'  (R)5'-CCA TCC AAT CGG TAG TAG CG-3' |
| TNF-α Mouse | | (F)5'-CAC CAC CAT CAA GGA CTC AA-3'  (R)5'-AGG CAA CCT GAC CAC TCT CC-3' |
| CD14 Mouse | | (F)5'-GGA AGC CAG AGA ACA CCA TC-3'  (R)5'-CCA GAA GCA ACA GCA ACA AG-3' |
| Myd88 Mouse | | (F)5'-AGA ACA GAC AGA CTA TCG GCT-3'  (R)5'-CGG CGA CAC CTT TTC TCA AT-3' |
| TGFβ1 Mouse | | (F)5'-ATT CCT GGC GTT ACC TTG-3'  (R)5'-CTG TAT TCC GTC TCC TTG GTT-3' |
| procollagen(I) Mouse | | (F)5'-TTC ACC TAC AGC ACG CTT GTG-3'  (R)5'-GAT GAC TGT CTT GCC CCA AGT T-3' |
